# Male polycystic ovarian syndrome phenotype: a meta-analysis of endocrine-metabolic dysregulation in fathers and brothers of PCOS-affected women

**DOI:** 10.1186/s12610-025-00290-1

**Published:** 2025-11-16

**Authors:** Kyana Jafarabady, Ida Mohammadi, Shahryar Rajai Firouzabadi, Fateme Mohammadifard, Sana Mohammad Soltani, Amirreza Paksaz, Mahsa Noroozzadeh, Fahimeh Ramezani Tehrani

**Affiliations:** 1 Reproductive Endocrinology Research Center, Research Institute for Endocrine Sciences, Shahid Beheshti University of Medical Sciences, Tehran, Iran; 2 Student Research Committee, School of Medicine, Shahid Beheshti University of Medical Sciences, Tehran, Iran; 3 Student Research Committee, School of Medicine, Alborz University of Medical Sciences, Karaj, Iran; 4 Foundation for Research & Education Excellence, Vestavia Hills, AL USA

**Keywords:** Polycystic ovary syndrome, Endocrine-metabolic dysregulation, Male equivalent of PCOS, Meta-analysis, Syndrome des Ovaires polykystiques, Dérégulation endocrino-métabolique, Équivalent masculin du SOPK, Méta-Analyse

## Abstract

**Background:**

Polycystic ovary syndrome (PCOS) is a common endocrine disorder in women with potential familial and genetic components. Emerging evidence suggests that male first-degree relatives (fathers and brothers) may exhibit endocrine and metabolic abnormalities similar to a “male equivalent” of PCOS, although the condition remains without clear diagnostic criteria. We conducted a systematic review and meta-analysis to investigate whether male relatives of women with PCOS show consistent patterns of metabolic and hormonal dysregulation.

**Results:**

A total of 21 studies met inclusion criteria, encompassing male first-degree relatives of women with PCOS with available data on metabolic, hormonal, and cardiovascular outcomes. Meta-analysis showed that male relatives had significantly higher fasting blood glucose (MD: 6.25; 95% CI: 1.36–11.14), body mass index (1.18; 0.35–2.02), triglycerides (17.82; 10.82–24.81), total cholesterol (18.63; 6.16–31.10), LDL-cholesterol (12.99; 1.27–24.71), and dehydroepiandrosterone sulfate (1.29; 0.66–1.92) compared with controls. They also exhibited higher prevalence of hypertension (OR: 1.88; 1.18–2.29), waist circumference > 90 cm (3.27; 1.18–9.08), and androgenetic alopecia (1.65; 1.04–2.60). Findings were consistent across studies, with low to moderate heterogeneity and minimal publication bias.

**Conclusion:**

Male first-degree relatives of women with PCOS demonstrate increased rates of metabolic abnormalities, hormonal imbalances, and androgenic features, supporting the concept of a male PCOS of equivalent. These findings underscore the familial nature of PCOS and highlight the need for improved diagnostic criteria and higher clinical awareness. Screening male relatives for metabolic and hormonal risk factors may help identify at-risk individuals and inform preventive interventions.

**Trial registration:**

IR.SBMU.ENDOCRINE.REC.1403.146.

## Résumé

**Contexte:**

Le syndrome des ovaires polykystiques (SOPK) est un trouble endocrinien courant chez les femmes avec des composantes familiales et génétiques potentielles. De nouvelles données suggèrent que les parents masculins au premier degré (pères et frères) peuvent présenter des anomalies endocriniennes et métaboliques similaires à un « équivalent masculin » de SOPK, bien que la maladie reste sans critères diagnostiques clairs. Nous avons réalisé une revue systématique et une méta-analyse pour déterminer si les parents masculins des femmes atteintes de SOPK présentaient des profiles cohérents de dérégulation métabolique et hormonale.

**Résultats:**

Au total, 21 études répondaient aux critères d’inclusion, à savoir des parents masculins au premier degré de femmes atteintes du SOPK avec des données disponibles sur les résultats métaboliques, hormonaux et cardiovasculaires. La méta-analyse a montré que les parents masculins avaient une glycémie à jeun significativement plus élevée (Différence de Moyennes : 6,25 ; IC95 % : 1,36-11,14), un indice de masse corporelle (1,18 ; 0,35-2,02), des triglycérides (17,82 ; 10,82 à 24,81), un cholestérol total (18,63 ; 6,16 à 31,10), un cholestérol LDL (12,99 ; 1,27 à 24,71) et le sulfate de déhydroépiandrostérone (1,29 ; 0,66 à 1,92) que les témoins. Ils présentaient également une prévalence plus élevée d’hypertension (OR : 1,88 ; 1,18-2,29), de tour de taille >90 cm (3,27 ; 1,18-9,08) et d’alopécie androgénique (1,65 ; 1,04-2,60). Les résultats étaient cohérents d’une étude à l’autre, avec une hétérogénéité faible à modérée et un biais de publication minimal.

**Conclusions:**

Les parents masculins au premier degré des femmes atteintes de SOPK présentent des taux accrus d’anomalies métaboliques, de déséquilibres hormonaux et de caractéristiques androgéniques, ce qui soutient le concept d’un équivalent masculin de SOPK. Ces résultats soulignent la nature familiale du SOPK, et font ressortir la nécessité d’améliorer les critères de diagnostic et celle d’accroître la sensibilisation clinique. Le dépistage des facteurs de risque métaboliques et hormonaux chez les parents masculins peut aider à identifier les personnes à risque et à les informer sur les interventions préventives.

## Introduction

Polycystic ovary syndrome (PCOS) is the most prevalent endocrine disorder affecting women of reproductive age. Based on the diagnostic criteria established by the National Institutes of Health (NIH), its prevalence is estimated to range between 6 and 9% across different populations worldwide [1]. PCOS is a heterogeneous clinical condition, and no single set of criteria can fully capture its complexity. However, according to the most widely accepted diagnostic guidelines, a diagnosis requires the presence of irregular menstrual cycles, hyperandrogenism (clinical or biochemical), and/or polycystic ovarian morphology on ultrasound [2]. The family clustering and genetic associations observed in PCOS suggest that the syndrome and its associated traits may have an inherited component [3].

While PCOS is generally defined as an endocrinopathy in women, observations of similar symptoms in men related to those with PCOS have resulted in the introduction of the term "Male equivalent of PCOS” [4]. Early-onset androgenetic alopecia (AGA), a key phenotypic feature, is often regarded as a hallmark manifestation of this condition [5]. Men with the male equivalent of PCOS may also exhibit reproductive and metabolic dysregulation similar to those observed in their female relatives [6]. Reproductive manifestations in men may include hormonal imbalances, such as elevated luteinizing hormone (LH) and dehydroepiandrosterone sulfate (DHEAS), accompanied by reduced levels of follicle-stimulating hormone (FSH) and sex hormone-binding globulin (SHBG) [7, 8]. Other reproductive manifestations include: sexual dysfunction [9] with hypothetical adverse effects on spermatogenesis [10]. Metabolic manifestations namely: High insulin resistance [11], hyperinsulinemia, dyslipidemia and increased risk of diabetes mellitus, hypertension and cardiovascular diseases have also been reported [12, 13].

The diagnostic criteria for PCOS have been a topic of debate since their inception [14]; This issue is even more pronounced in the male equivalent of PCOS, as its definitions, clinical manifestations, diagnostic criteria, and distinction from metabolic syndrome remain unclear [12, 15].To the best of our knowledge, no studies have fully examined the male equivalent of PCOS and its associated signs and symptoms in first-degree male relatives of women with the syndrome. Therefore, this study aims to conduct a systematic review and meta-analysis to evaluate the various clinical, hormonal, and metabolic features of male equivalent of PCOS.

### Patients and methods

This systematic review and meta-analysis was conducted in accordance with the Preferred Reporting Items for Systematic Reviews and Meta-Analyses (PRISMA) guidelines [16]. This study is registered in PROSPERO with CRD42024608920 registration number. The proposal for this research was approved by the Ethics Committee of the Infertility and Reproductive Health Research Center (IRHRC), Shahid Beheshti University of Medical Sciences (IR.SBMU.ENDOCRINE.REC.1403.146).

#### Search strategy

A comprehensive search of PubMed, Scopus, and Web of Science was conducted until July 2024 using keywords and MeSH terms related to “polycystic ovary syndrome” and “first-degree male relative.” The exact search strategies for each database are provided in Table 1. No restrictions were applied regarding publication year, and only articles published in English were included.

**Table 1:** Detailed Search Strategies Applied for Online Database Queries

Database	Search String
MEDLINE (via PubMed)	(Polycystic Ovary Syndrome[MeSH Terms] OR PCOS OR "polycystic ovary syndrome" OR "polycystic ovarian syndrome") AND ("first-degree relatives"[Title/Abstract] OR “family members”[Title/Abstract] OR “family history”[MeSH Terms] OR “father”[Title/Abstract] OR “mother”[Title/Abstract] OR “daughter”[Title/Abstract] OR “son”[Title/Abstract] OR “brother”[Title/Abstract] OR “sister”[Title/Abstract] OR “sibling”[Title/Abstract] OR “offspring”[Title/Abstract] OR “relatives”[Title/Abstract])
Web of Science	TS = ("polycystic ovary syndrome" OR PCOS OR "polycystic ovarian syndrome") AND TS = ("first-degree relatives" OR “family members” OR “family history” OR father OR mother OR daughter OR son OR brother OR sister OR sibling OR offspring OR relatives)
Scopus	TITLE-ABS-KEY("polycystic ovary syndrome" OR PCOS OR "polycystic ovarian syndrome") AND TITLE-ABS-KEY("first-degree relatives" OR “family members” OR “family history” OR father OR mother OR daughter OR son OR brother OR sister OR sibling OR offspring OR relatives)

#### Eligibility criteria

Studies were included if they met the following PECOS criteria: Population (P): male first-degree relatives (sons, fathers, or brothers) of women with a confirmed diagnosis of PCOS; Exposure (E): not applicable; Comparator (C): matched healthy male controls; Outcomes (O): PCOS-related signs and outcomes, including metabolic disorders, gonadotropin levels (LH and FSH) anti-Müllerian hormone (AMH) levels, androgen levels (testosterone or DHEAS), dermatological manifestations (e.g., acne vulgaris, alopecia, hair loss), infertility parameters (sperm count, motility, morphology), lipid profile (low-density lipoprotein Cholesterol (LDL-C), high-density lipoprotein Cholesterol (HDL-C), total cholesterol, triglycerides), blood pressure (systolic and diastolic), hypertension, and cardiovascular diseases (e.g., stroke); Study design (S): observational case–control studies.

Studies were excluded if they investigated second-degree male relatives of women with PCOS, lacked a healthy control group, or were case reports, case series, controlled trials, or review articles.

#### Screening process

Screening was conducted in two phases by two independent reviewers (S.S. and A.P.) under the supervision of a senior reviewer (I.M.). The first phase involved title and abstract screening, followed by full-text retrieval and assessment in the second phase. Any discrepancies between the reviewers were resolved through discussion with the third reviewer.

#### Data extraction

Data were extracted independently by two reviewers (I.M. and S.R.) using a pre-designed spreadsheet that included the following variables: author, year of publication, number and type of first-degree male relatives, and number of matched healthy controls. Any discrepancies were resolved through discussion with an independent reviewer (K.J.)

#### Outcomes

Our outcomes of interest encompassed PCOS-related signs and sequelae, including continuous measures such as androgen levels (AD), body mass index (BMI), cholesterol, DHEAS, fasting blood glucose, FSH, LH, HDL, Homeostatic Model Assessment of Insulin Resistance (HOMA-IR), total testosterone, and urinary testosterone (uT), as well as categorical outcomes such as androgenetic alopecia, diabetes mellitus, hypertension, heart disease, obesity, and stroke.

#### Quality assessment

The Newcastle–Ottawa Scale (NOS) [17] was used to assess risk of bias by two independent reviewers. NOS consists of three domains, selection, comparability, and exposure and provides a maximum score of 9. If a study obtained ≥ 70% of the highest level of the Newcastle–Ottawa scale, it was considered as high quality, those with 40–70% as moderate, those with 20–40% as low, and those with < 20% as very low quality.

#### Search results, study selection, study characteristics, and quality assessment

**Fig. 1:**
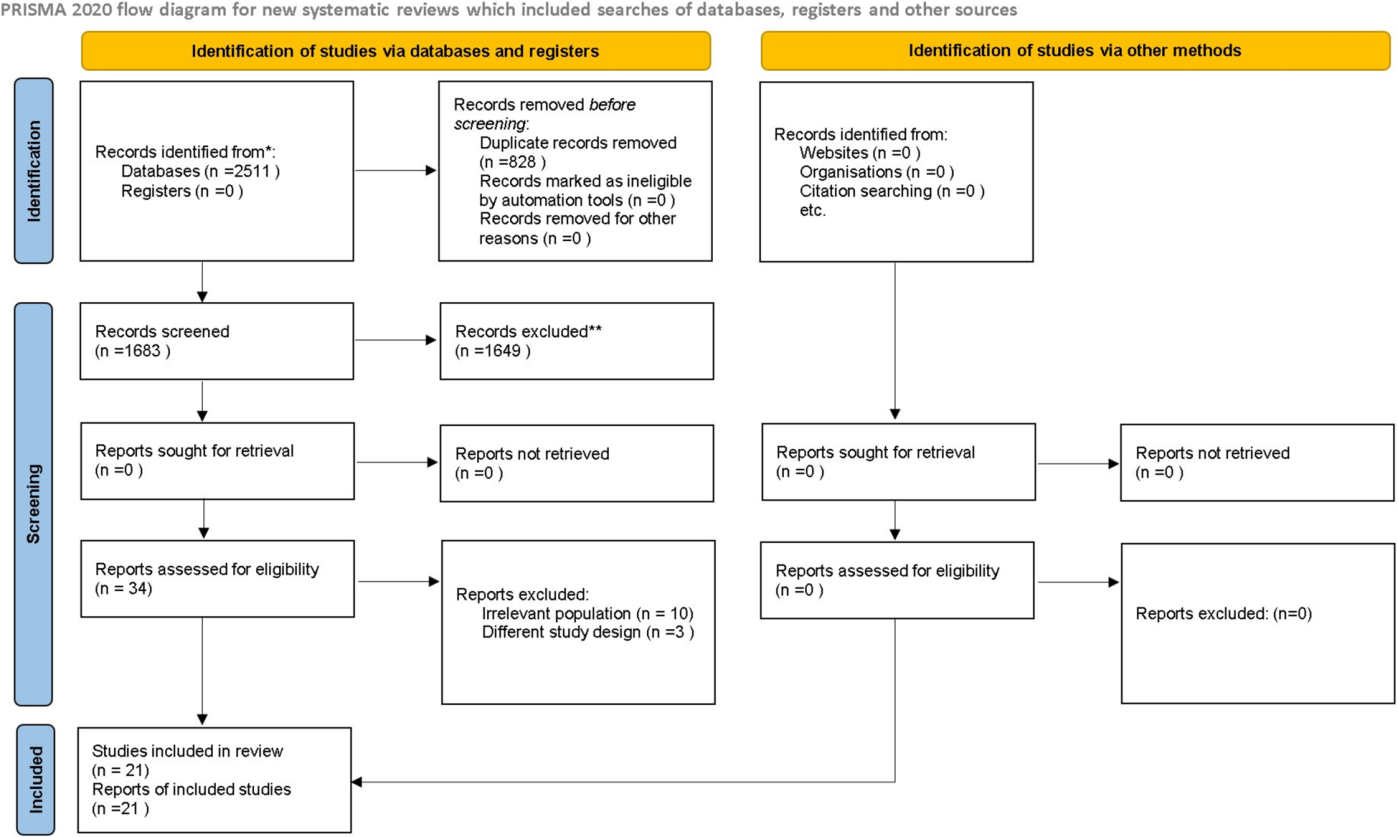
PRISMA flow chart

A total of 2,511 records were identified through databases and other sources. After removing duplicates, 1,683 articles were screened by title and abstract, of which 34 were retrieved for full-text assessment. Ultimately, 21 studies met the inclusion criteria (Fig. 1). Among these, one was a cohort study, two were cross-sectional studies, and 18 were case–control studies. The characteristics of the included studies are summarized in Table 2. Overall, the majority of studies were assessed as high quality, while four were rated as moderate quality [18–21]. No included study was assessed as being of low or very low quality (Table 3).

**Table 2:** Characteristics and Key Findings of Included Studies

Author	Year	Number of male relatives of females with PCOS	Number of male relatives of females without PCOS	Relation of male relatives to females with PCOS	Main findings
Hunter et al. [22]	2007	103	201	Father, Brother	No significant differences were identified in the prevalence of heart disease, stroke, or diabetes between fathers of women with PCOS and control fathers. In contrast, brothers of women with PCOS demonstrated significantly elevated cholesterol levels and a higher prevalence of weight-related issues compared to control brothers
Coviello et al. [23]	2009	269	1735	Father, Brother	The prevalence of metabolic syndrome was significantly greater in male relatives of women with PCOS compared to the control group. Additionally, fathers and brothers of PCOS patients exhibited higher body mass index and increased mean waist circumference (P < 0.0001). Fathers with metabolic syndrome demonstrated lower total testosterone levels after adjustment for body mass index (p = 0.02). Similarly, brothers with metabolic syndrome showed reduced urinary testosterone levels after adjustment for both body mass index and age
Lenarcik et al. [24]	2011	42	30	Brother	Glucose levels, insulin levels at 120 min, total cholesterol, and low-density lipoprotein concentrations were significantly higher in brothers of women with PCOS compared to control counterparts. However, no significant differences were observed between the two groups in insulin sensitivity and resistance indices, high-density lipoprotein, or triglyceride levels
Lenarcik et al. [25]	2011	42	30	Brother	DHEA-S levels were markedly higher in brothers of women with PCOS (p < 0.001). However, no statistically significant differences were observed in total testosterone and androstenedione levels. Furthermore, no variations in hormonal profiles were reported in brothers of women with PCOS in relation to balding
Yildiz et al. [26]	2003	49	27	Father, Brother	No significant differences were observed in hormonal parameters, including LH, FSH, total testosterone, and DHEA-S, between brothers and fathers of women with PCOS and the control group. The insulin area under the curve during the oral glucose tolerance test was elevated in the brother group; however, fasting insulin levels and HOMA-IR showed no significant differences between the groups
Liu et al. [27]	2014	29	18	Brother	Brothers of women with PCOS exhibited significantly higher FSH and lower DHEA-S levels compared to control men. However, no statistically significant differences were observed in LH, total testosterone, and androstenedione levels
Legro et al. [28]	2002	119	68	Brother	Substantially higher DHEA-S levels were reported in brothers of the PCOS group compared to controls after adjusting for age. However, no significant differences were observed in other hormonal parameters, including total testosterone, urinary testosterone, LH, and FSH
Legro et al. [29]	2010	193	82	Father, Brother	No significant association was observed between phenotype and birthweight in either female or male family members of women with PCOS
Lunde et al. [30]	1989	279	139	Father, Brother	A significantly higher prevalence of baldness was observed in brothers and fathers of women with PCOS compared to first-degree male controls (p < 0.01). Notably, female relatives of PCOS patients exhibited a greater incidence of PCOS-related symptoms compared to female controls
Torchen et al. [21]	2016	129	88	Father, Brother	Higher levels of LH, FSH, and anti-Müllerian hormone were observed in brothers and fathers of women with PCOS compared to both younger and older control men. However, estradiol, estrone, testosterone, and inhibin B levels did not differ significantly between the groups. Fathers of women affected by PCOS demonstrated an increased susceptibility to heart disease and stroke relative to control men. Furthermore, the prevalence of cardiovascular disease and hypertension was elevated in mothers of women with PCOS
Davies et al. [31]	2011	41	674	Father	Elevated levels of LH, FSH, and anti-Müllerian hormone were detected in brothers and fathers of women with PCOS compared to both younger and older control men. Conversely, levels of estradiol, estrone, testosterone, and inhibin B did not differ significantly between groups. Fathers of women with PCOS exhibited an increased susceptibility to heart disease and stroke relative to control men. Additionally, the prevalence of cardiovascular disease and hypertension was higher among mothers of women with PCOS
Moradi et al. [32]	2010	34	34	Father	The prevalence of metabolic syndrome and hypertension was significantly higher in fathers of patients diagnosed with PCOS. Elevated levels of fasting blood glucose, low-density lipoprotein, total cholesterol, triglycerides, and testosterone were observed in fathers of women with PCOS compared to the control group. No significant differences in insulin resistance, assessed by HOMA-IR or the quantitative insulin sensitivity check index, were reported between the two groups
Subramaniam et al. [33]	2019	41	41	Brother	A statistically significant increase in DHEA-S levels, elevated blood pressure, higher HOMA-IR, and fasting insulin levels were observed in male siblings of individuals diagnosed with PCOS. The prevalence of metabolic syndrome was higher among fathers of affected individuals. No significant differences were identified in low-density lipoprotein, high-density lipoprotein, or triglyceride levels between the groups
Krysiak et al. [34]	2021	24	26	Brother	In brothers of patients with PCOS, significant elevations in HOMA-IR, androstenedione, and triglyceride levels were observed compared to the control group. Conversely, the control group exhibited higher levels of high-density lipoprotein and urinary testosterone. No significant differences were detected between groups in low-density lipoprotein, total cholesterol, or total testosterone levels
Sam et al. [19]	2008	169	169	Brother	Brothers of women with PCOS exhibited elevated levels of total cholesterol, low-density lipoproteins, triglycerides, fasting insulin, and insulin resistance compared to control men. Additionally, positive correlations were observed between the cholesterol, triglyceride, and insulin levels of brothers and those of their sisters with PCOS
Taylor et al. [35]	2011	180	NP	Father	Fathers of women with PCOS exhibited a higher prevalence of myocardial infarction and stroke compared to the control population, along with an increased 10-year risk of coronary heart disease. However, no significant elevation in cardiovascular events or 10-year risk was observed in the women with PCOS or their mothers
Vipin et al. [36]	2016	41	42	Father	Parents of women with PCOS exhibited increased carotid intima-media thickness and reduced flow-mediated dilatation compared to controls, alongside elevated blood pressure, low-density lipoprotein, and glucose levels. They also demonstrated a higher prevalence of cardiovascular risk factors, including hypertension, diabetes, abdominal obesity, and a family history of coronary artery disease. Fathers showed a higher prevalence of diabetes, whereas mothers presented comparable levels of other risk factors
Govind et al. [18]	1999	51	21	Fathers, brothers	Among 71 siblings of PCOS probands, 39 were affected, yielding a segregation ratio of 55%, which supports an autosomal dominant inheritance pattern for PCOS and premature male pattern baldness. In contrast, only 3 of 24 control siblings were affected, indicating a segregation ratio significantly inconsistent with autosomal dominant inheritance
Villeneuve et al. [37]	2009	11	21	Brothers	Low urinary clearance of D-chiro-inositol was strongly associated with hyperinsulinemia in all men. Brothers of women with PCOS exhibited greater insulin resistance and elevated plasma D-chiro-inositol levels. Additionally, these brothers demonstrated a reduced ratio of urinary clearance of D-chiro-inositol to myo-inositol
Baillargeon et al. [38]	2007	17	28	Brothers	Brothers of women with PCOS exhibited comparable body mass index, waist circumference, body fat percentage, and blood pressure relative to controls but demonstrated elevated levels of triacylglycerol, plasminogen activator inhibitor-1, factor VIII, and glucose. Insulin sensitivity was reduced by 38% in these brothers, primarily due to a 65% decrease in insulin-stimulated non-oxidative carbohydrate metabolism. These differences remained significant after adjusting for age and body mass index and were more pronounced among obese brothers
Sam et al. [20]	2009	23	23	Brothers	Brothers of women with PCOS exhibited a significantly reduced disposition index and elevated glucose effectiveness compared to control men. These findings suggest that brothers of women with PCOS may experience pancreatic beta-cell dysfunction and possess an increased risk for type 2 diabetes

*Abbreviations*: *PCOS* Polycystic ovary syndrome, *DHEA-S* Dehydroepiandrosterone sulfate, *LH* Luteinizing hormone, *FSH* Follicle stimulating hormone, *HOMA-IR* Homeostatic model assessment for insulin resistance,NP normal population

**Table 3:** Quality assessment

Case control studies											
Authors	Is case definition adequate?	Representativeness of the cases	Selection of controls	Definition of controls	Comparability	Assessment of the exposure	Same method ascertainment for case and controls?	Non-Response Rate	Total score		assessment
Hunter et al. [34]	1	1	1	1	1	0	1	1	7		Good
Yildiz et al. [24]	1	1	0	1	0	1	1	1	6		Good
Torchen et al. [39]	1	1	0	0	1	1	1	1	6		Fair
Subramaniam et al. [40]	1	1	0	0	2	1	1	1	7		Good
Baillargeon et al. [31]	1	0	1	1	1	1	1	1	7		Good
Govind et al. [19]	1	0	1	0	0	1	1	1	5		Fair
Sam et al. [19]	0	1	1	1	0	1	0	1	5		Fair
Sam et al. [21]	0	1	1	1	0	1	0	1	5		Fair
Vipin et al. [26]	1	0	1	1	0	1	1	1	6		Good
Legro et al. [23]	1	1	1	1	1	1	1	1	8		Good
Legro et al. [41]	1	1	1	1	1	1	1	1	8		Good
Liu et al. [42]	1	1	1	1	1	1	1	1	8		Good
Lunde et al. [43]	1	1	1	1	1	1	1	1	8		Good
Moradi et al. [33]	1	1	1	1	1	0	1	1	7		Good
Krysiak et al. [25]	1	1	1	1	1	1	1	1	8		Good
Villeneuve et al. [36]	1	1	1	1	1	1	1	1	8		Good
Taylor et al. [44]	1	1	1	1	1	1	1	1	8		Good
Lenarcik et al. [32]	1	1	1	1	1	1	1	1	8		Good
Lenarcik et al. [45]	1	1	1	1	1	1	1	1	8		Good
cross sectional studies											
Authors	Representive of the cases	Sample size	Non-Response rate	Ascertainment of the screening/surveillance tool	Comparability	Assessment of the outcome	Statistical test	Total score	assessment		
Coviello et al. [27]	1	1	0	2	1	0	1	6	Good		
cohort studies											
Author	1.Representativeness of the exposed cohort	2. Selection of the non-exposed cohort	3. Ascertainment of exposure	4.Demonstration that outcome of interest was not present at start of study	5 Comparability	6. Assessment of the outcome	7. Was follow-up long enough for outcomes to occur	8.Adequacy of follow-up of cohorts	Total score	Assessment	
Davies et al. [22]	1	0	1	0	2	1	1	1	7	Good	

^a^The detailed questions are available in: https://www.nhlbi.nih.gov/health-topics/study-quality-assessment-tools

#### Statistical analyses

All statistical analyses were performed using R software (version 4.3.2) with the meta package. Only means and standard deviations or the number of events and total participants were used for data entry. Effect sizes were expressed as mean differences (MD) or odds ratios (OR) with corresponding 95% confidence intervals (95% CI). A random-effects model was employed to account for differences in participant age and variations in outcome measurement scales across studies. Heterogeneity was evaluated using the I^2^ statistic, with I^2^ > 50% considered indicative of significant heterogeneity [39]. A *p*-value of < 0.05 was considered statistically significant. Heterogeneity was further explored through subgroup analyses based on the type of male relative (brothers versus fathers), with a chi-squared *p*-value of < 0.1 indicating significant differences between subgroups.

## Results

### Glycemic indices

Thirteen studies investigated various glycemic parameters, including fasting Blood Sugar (FBS) level, HOMA-IR, and diabetes status [19, 20, 22, 25, 26, 31–34, 36–38, 40]. Six studies showed a significant statistical difference in FBS levels between first-degree male relatives of women with PCOS and control men [19, 24, 32, 36–38], whereas others reported no differences in FBS between these groups [20, 33, 34]. In the case of HOMA-IR, three studies showed significant differences between the two groups [33, 34, 36]. Only one study showed a higher risk of diabetes in relatives of women with PCOS [36].

**Fig. 2:**
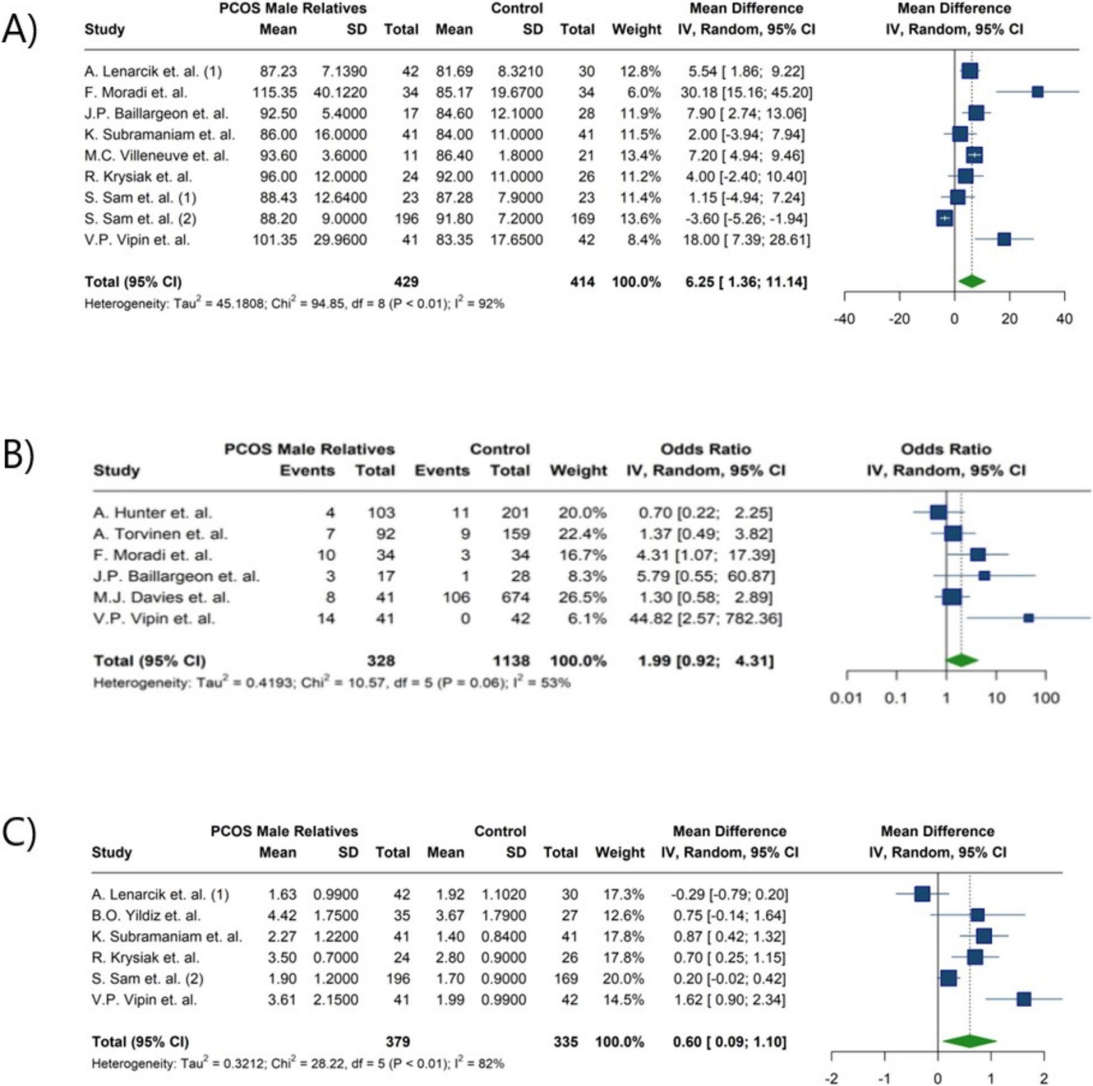
Forest plot of meta-analysis of glycemic indices: **A**) Fasting blood sugar (mg/dl), **B**) Diabetes mellitus, **C**) Homeostasis model assessment for insulin resistance

**Fig. 3:**
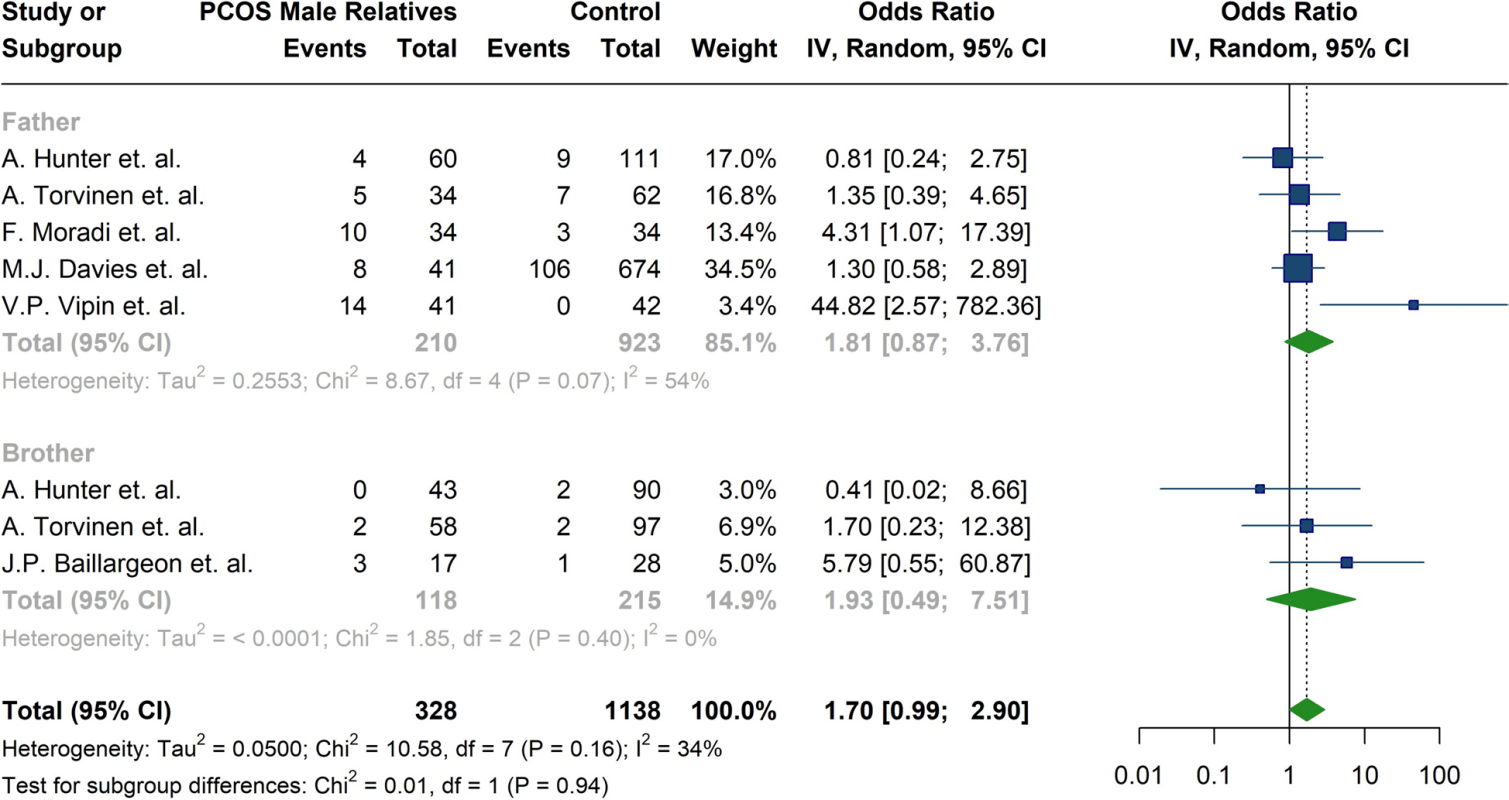
Forest plot of the subgroup analysis comparing diabetes prevalence between fathers and brothers of women with PCOS

Regarding diabetes, no statistically significant differences were identified between first-degree male relatives of women with PCOS and control men (OR: 1.99; 95%CI: [0.92; 4.31]; I^2^: 53%). In contrast, a significant statistical difference was revealed in FBS levels between the groups (MD: 6.25; 95%CI: [1.36; 11.14]; I^2^: 92%). For insulin resistance, as measured by HOMA-IR, no notable difference was observed (MD:0.60; 95%CI: [0.09; 1.10]; I^2^: 82%) (Fig. 2) (Table 4). Subgroup analysis for diabetes between fathers and brothers also showed no statistically significant differences (Table 5) (Fig. 3).

**Table 4:** Summary of meta-analysis findings

Outcomes	Number of studies	Odd Ratio [95% CI]	*P*-Value	I^2^ [95%CI]
Diabetes	6	1.9938 [0.9217; 4.3133]	0.0796	52.7% [0.0%; 81.1%]
Hypertension	6	1.8764 [1.1783; 2.9882]	**0.0080**	35.7% [0.0%; 74.3%]
Obesity	3	3.2745 [1.1811; 9.0785]	**0.0226**	66.2% [0.0%; 90.3%]
Stroke	2	1.8503 [0.1877; 18.2450]	0.5982	87.6% [51.6%; 96.8%]
Heart disease	3	1.6869 [0.9453; 3.0101]	0.0768	57.8% [0.0%; 88.0%]
Baldness	5	1.6465 [1.0428; 2.5995]	**0.0324**	0.0% [0.0%; 79.2%]
Outcomes	Number of studies	Mean Difference [95% CI]	*P* value	I^2^ [95%CI]
BMI (kg/m^2^)	14	1.1820 [0.3489; 2.0151]	**0.0054**	83.8% [74.2%; 89.8%]
FBS (mg/dl)	9	6.2470 [1.3560; 11.1379]	**0.0123**	91.6% [86.2%; 94.8%]
LDL-C (mg/dl)	7	12.9880 [1.2663; 24.7096]	**0.0299**	82.0% [64.0%; 91.0%]
HDL -C(mg/dl)	8	−2.0156 [−4.2811; 0.2499]	0.0812	55.3% [1.2%; 79.8%]
Triglyceride (mg/dl)	8	17.8150 [10.8182; 24.8119]	**< 0.0001**	16.2% [0.0%; 59.2%]
Total cholesterol (mg/dl)	7	18.6318 [6.1618; 31.1019]	**0.0034**	73.2% [42.5%; 87.5%]
HOMA-IR	6	0.5988 [0.0932; 1.1044]	**0.0203**	82.3% [62.4%; 91.6%]
FSH (IU/L)	4	0.4676 [−1.0257; 1.9608]	0.5394	79.0% [43.9%; 92.2%]
LH (IU/L)	4	0.3995 [−1.3298; 0.5308]	0.3999	82.0% [53.4%; 93.0%]
Total testosterone (nmol/L)	8	−1.1110 [−2.5570; 0.3351]	0.1321	57.9% [7.8%; 80.8%]
Bioavailable testosterone (uT) (nmol/L)	3	−0.2769 [−1.7672; 1.2134]	0.7158	69.3% [0.0%; 91.1%]
Androstenedione (nmol/L)	4	0.0522 [−0.6733; 0.7778]	0.8878	56.9% [0.0%; 85.7%]
DHEAS (µmol/l)	9	1.2898 [0.6553; 1.9243]	**< 0.0001**	41.6% [0.0%; 73.1%]

*Abbreviations*:*95% CI* 95% confidence interval,*BMI* Body mass index,*kg/m*^*2*^ kilogram per square meter, *FBS* Fasting blood sugar, *mg/dl* Milligrams Per Deciliter, *LDL-C* Low density lipoprotein cholesterol, *HDL-C* High density lipoprotein cholesterol, *HOMA-IR* Homeostatic model assessment-insulin resistance, *FSH* Follicle-stimulating hormone, *IU/l* International units per liter, *LH* Luteinizing hormone,*nmol/L* nanomoles per liter, *DHEA-S* Dehydroepiandrosterone-sulfate, *µmol/L* Micromoles per liter

**Table 5:** Summary of Meta-Analysis Findings Stratified by Relative Status in Male Relatives of Women with PCOS (Fathers and Brothers)

Outcomes	Subgroups	Number of studies	Odd Ratio [95% CI]	Between groups *P*-Value^a^	I^2^
Diabetes	Fathers	5	1.8104 [0.8717; 3.7601]	0.9370	53.9%
	Brothers	3	1.9268 [0.4945; 7.5074]		0.0%
Hypertension	Fathers	5	1.8087 [1.0583; 3.0914]	0.5932	33.1%
	Brothers	3	2.3197 [1.1079; 4.8568]		0.0%
Outcomes	Subgroups	Number of studies	Mean Difference [95% CI]	Between groups *P*-Value^a^	I^2^
BMI (kg/m^2^)	Fathers	5	1.5161 [0.0328; 2.9994]	0.6207	82.8%
	Brothers	13	1.0785 [0.1821; 1.9750]		79.5%
Total cholesterol (mg/dl)	Fathers	3	23.1964 [−4.9603; 51.3532]	0.4955	88.8%
	Brothers	5	13.2137 [7.6137; 18.8136]		0.0%
FSH (IU/l)	Fathers	2	1.9935 [−0.6520; 4.6390]	0.1478	47.6%
	Brothers	4	−0.1151 [−1.1897; 0.9595]		65.6%
Total testosterone (nmol/L)	Fathers	2	−1.8745 [−5.4847; 1.7356]	0.5561	56.5%
	Brothers	8	−0.7056 [−2.1602; 0.7490]		55.4%

*Abbreviations*: *95% CI* 95% confidence interval, *BMI* Body mass index, *FSH* Follicle stimulating hormone, *kg/m*^*2*^ kilogram per square meter, *mg/dl* Milligrams Per Deciliter, *IU/l* International units per liter, *nmol/L* nanomoles per liter/

^a^The results obtained through analysis using the chi-squared test

In the article conducted by Moradi et al. [46] no statistically significant disparity was identified for type 2 diabetes. Similar findings were reported by Davis et al. [31] and Hunter et al. [22] concerning diabetes in fathers of women with PCOS against control men. Conflicting findings for HOMA-IR were observed, as Subramaniam et al. [33] and Krysiak et al. [34] found statistically higher HOMA-IR in brothers of women with PCOS compared with controls. Other studies reported no significant differences in HOMA-IR and QUICKI between male relatives of women diagnosed with PCOS and control men [24, 26, 46]. Regarding FBS levels, Moradi et al. [46] reported elevated FBS concentrations in fathers of women with PCOS, similarly, Lenarcik et al. [24] presented similar findings among brothers. In contrast, two studies have reported opposite results [33, 34].

### Obesity parameters

**Fig. 4:**
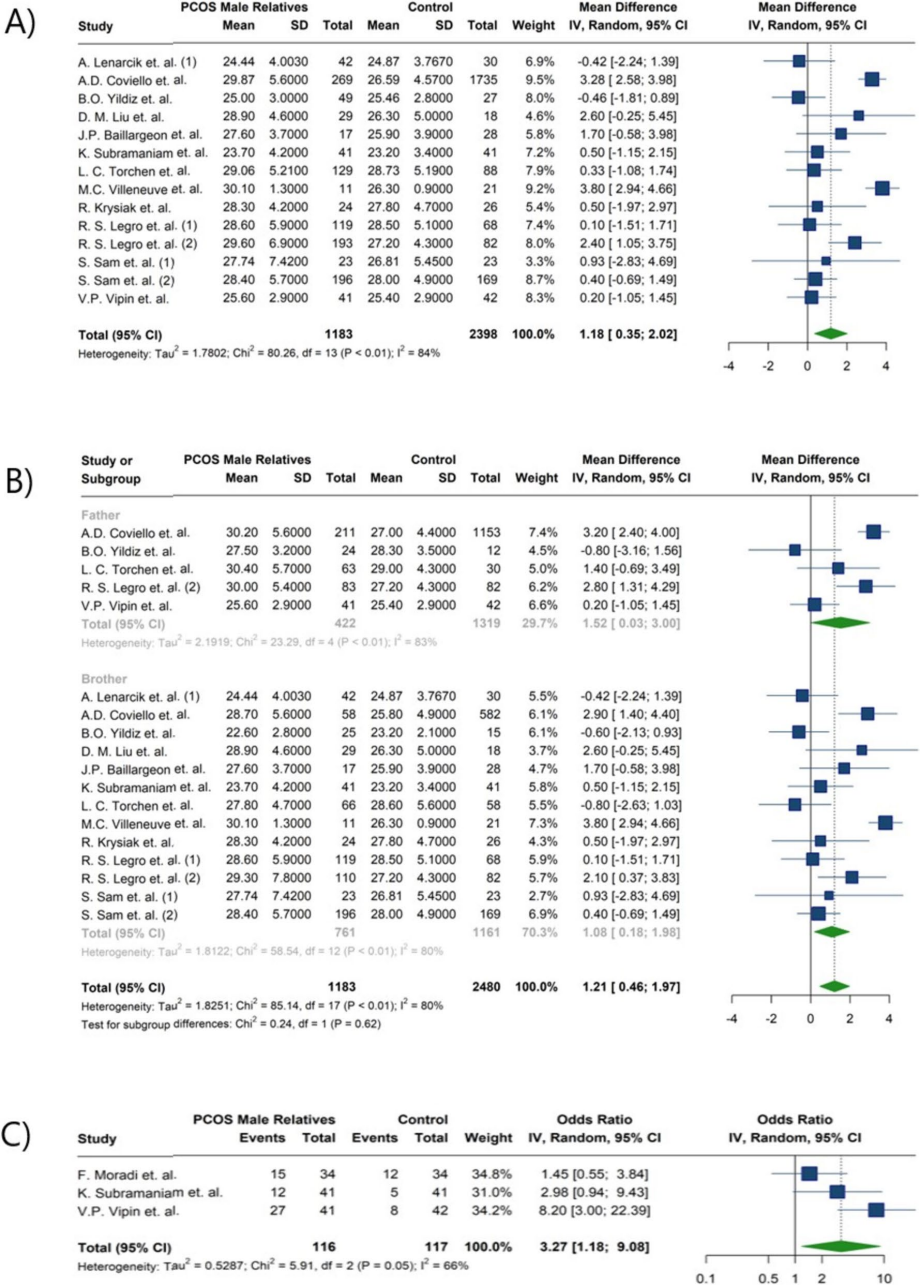
Forest plot presenting the meta-analysis of obesity-related parameters in male relatives of women with PCOS compared to control individuals.: **A**) Body mass index (kg/m^2^), **B**) the subgroup analysis comparing BMI between fathers and brothers of women with PCOS, **C**) prevalence of patients with waist circumference exceeding 90 cm

Fifteen studies evaluated obesity and BMI [19–21, 23, 24, 26–29, 32–34, 36–38]. One study reported a higher risk of obesity in relatives of PCOS patients [36], and three reported significant differences in BMI between these two groups [23, 29, 37]. In the analysis of obesity, data from three studies using the same definition of obesity showed a significant difference in waist circumference exceeding 90 cm between groups (OR: 3.27; 95%CI: [1.18; 9.08]; I^2^: 66%). Male relatives of women afflicted with PCOS had higher BMI compared to the control group (MD: 1.18; 95%CI: [0.35; 2.02]; I^2^: 84%). In subgroups analysis, fathers of women with PCOS have 1.52 kg/m^2^ higher BMI than control men (MD: 1.52; 95%CI: [0.03; 3.00]; I^2^: 83%), while brothers had 1.08 kg/m^2^ higher BMI compared with control group (MD: 1.08; 95%CI: [0.18; 1.98]; I^2^: 80%) (Fig. 4) (Table 4).

Regarding the obesity, Hunter et al. [22] used weight problem as the definition of obesity and did not observe significant differences between groups. Using the same WC cutoff (> 90 cm), Moradi et al. [46] found no significant differences between father of women with PCOS and controls, while Subramaniam et al. [33] indicated an increased prevalence of obesity in brothers. Vapin et al. reported significantly higher abdominal obesity (WC > 90 cm) in fathers of women with PCOS [36]. Most studies did not show significant differences in BMI between groups [21, 24, 26–29, 33, 34], In contrast Coviello et al. [23] identified higher BMI in first-degree male relatives of women with PCOS compared to controls.

### Lipid profiles

Lipid profiles, including TG, LDL-C, HDL-C, and Total cholesterol, were evaluated in nine studies [19, 24, 29, 32–34, 36–38]. Four studies reported significant differences in total cholesterol levels between the two groups [19, 24, 29, 32], and three revealed significant differences in TG level [32, 34, 38]. Only one study showed significant differences in LDL-C levels [32], and three reported significant differences in HDL-C levels [32, 34, 37].

**Fig. 5:**
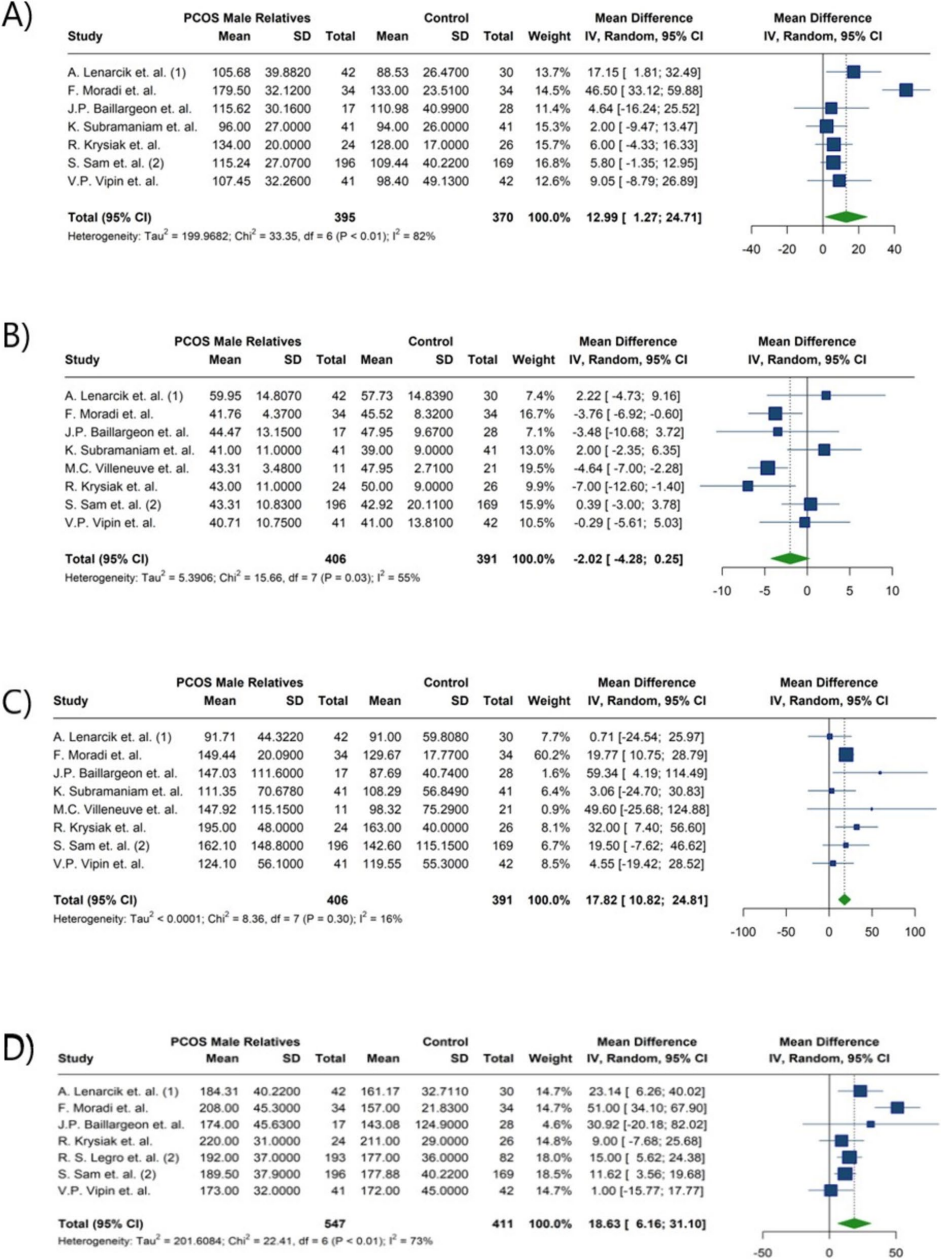
Forest plot presenting the meta-analysis of lipid profiles in male relatives of women with PCOS compared to control individuals. **A**) Low-density lipoprotein (mg/dl), **B**) High-density lipoprotein (mg/dl), **C**) Triglycerides (mg/dl), **D**) Cholesterol (mg/dl)

**Fig. 6:**
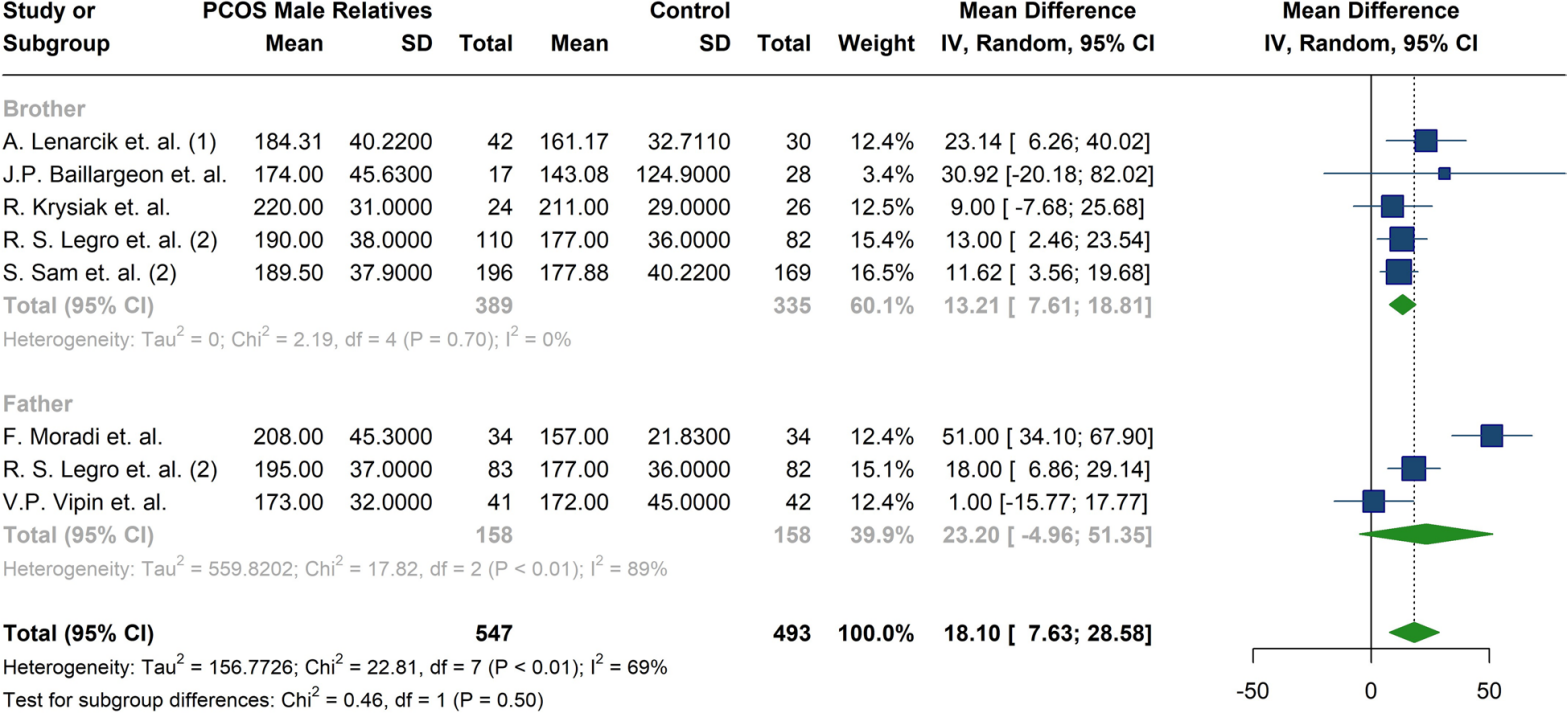
Forest plot of the subgroup analysis comparing cholesterol level (mg/dl) between fathers and brothers of women with PCOS

The meta-analysis of lipid profiles showed that male parents of women with PCOS had a statistically significant 17.82 mg/dl increase in TG levels compared to male controls (MD: 17.82; 95%CI: [10.82; 24.81]; I^2^: 16%). Similarly, males in experimental group had as 18.63 mg/dl increase in total cholesterol levels (MD: 18.63; 95%CI: [6.16; 31.10]; I^2^: 73%) (Fig. 5) (Table 4); After subgroup meta-analysis, this association remained significant for brothers (MD:13.21; 95%CI: [7.61; 18.81]; I^2^: 0%) but not for fathers (MD:23.20; 95%CI: [−4.96; 51.35]; I^2^: 89%) (Table 5) (Fig. 6). Lower HDL-C and higher LDL-C levels were observed in the male experimental group. The reduction in HDL did not reach statistical significance compared to controls, but the increase in LDL-C was statistically significant (HDL-C MD: −2.02; 95%CI: −4.28; 0.25; I^2^: 55% and LDL-C MD: 12.99; 95% CI:1.27; 24.71; I^2^: 82%) (Fig. 5) (Table 4). In the study by Moradi et.al. [46], levels of LDL-C, HDL-C, TG, and Total cholesterol showed significant differences between the two groups. Lenarcik et al. [24] revealed that LDL-C and Total cholesterol levels were elevated in brothers of women with PCOS. Other studies did not find significant distinctions between groups [29, 34]. Marked differences in HDL and TG levels were reported by Krysiak et al. [34]. while other studies presented conflicting findings [24, 33]. In the meta-analysis by Yilmaz et al. [41], significantly higher total cholesterol, LDL-C, and TG levels were observed in first-degree female relatives of women with PCOS. Our meta-analysis revealed similar findings, showing higher total cholesterol and TG levels in male relatives of women with PCOS.

### Hormonal parameters

**Fig. 7:**
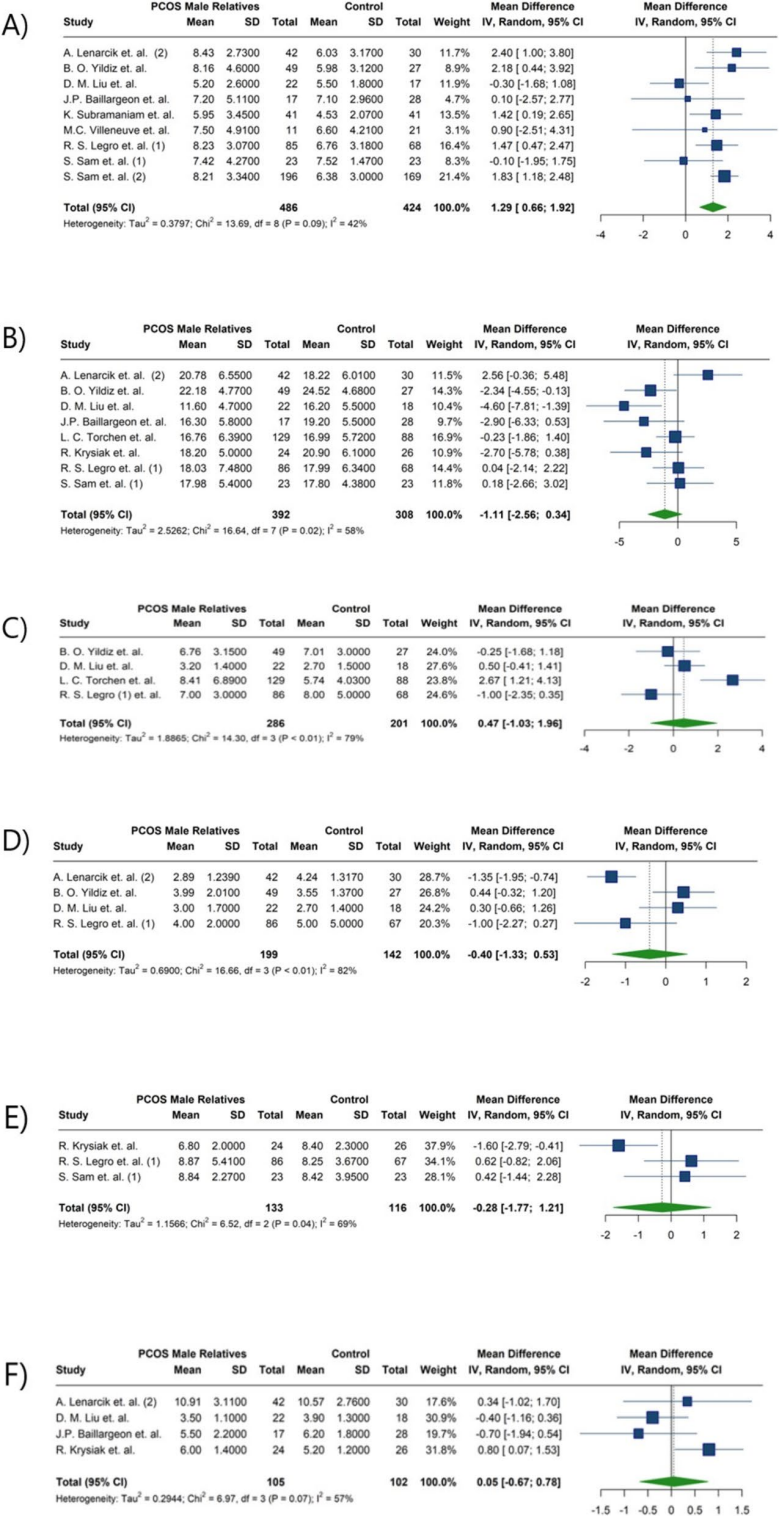
Forest plot of meta-analysis of hormonal parameters in male relatives of women with PCOS compared to control individuals. **A** Dehydroepiandrosterone sulfate(µmol/l), **B**) Total testosterone (nmol/L), **C**) Follicle-stimulating hormone (IU/L), **D**) Luteinizing hormone (IU/L), **E**) Bioavailable testosterone(nmol/L)

**Fig. 8:**
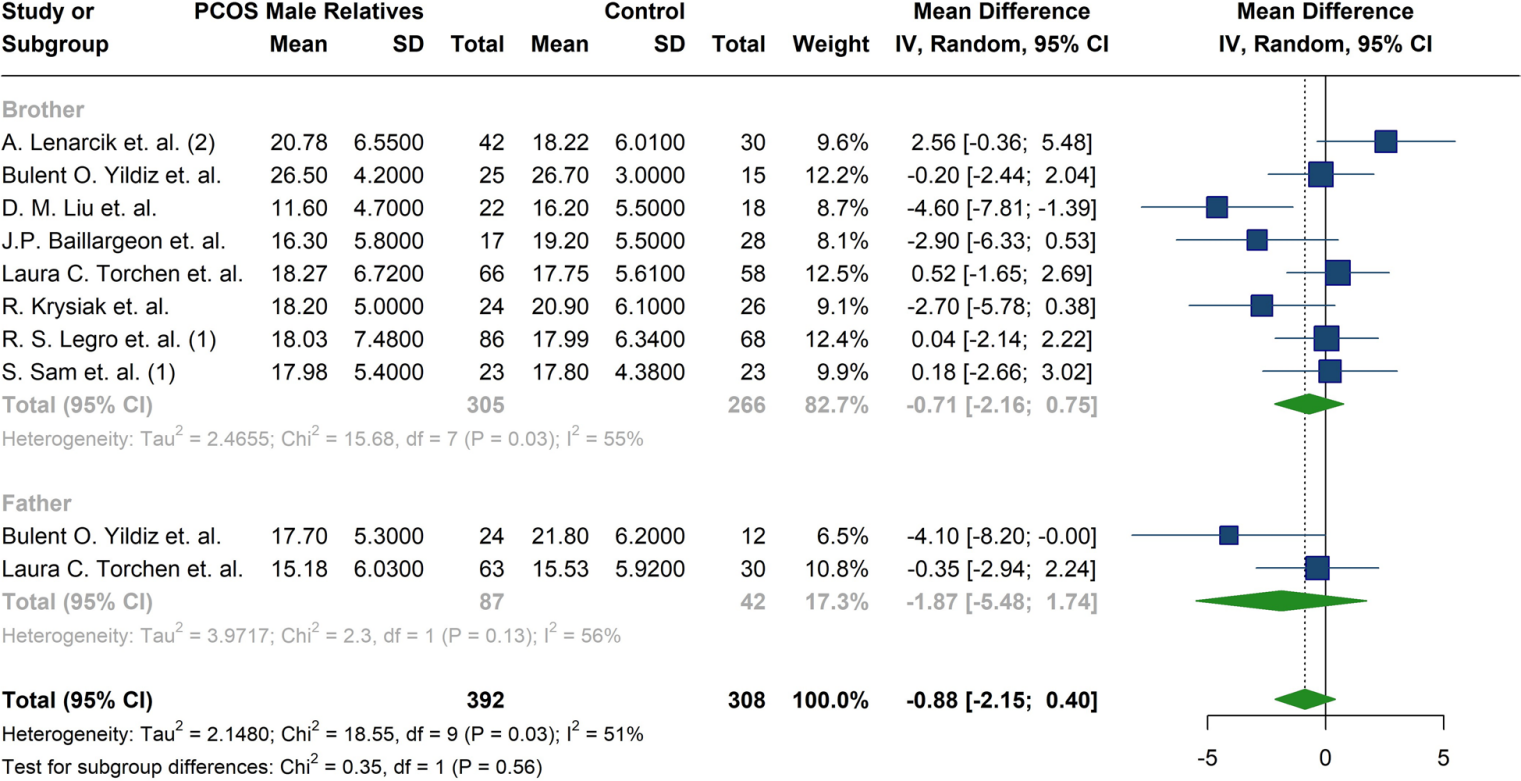
Forest plot of the subgroup analysis comparing Total testosterone (nmol/L) between fathers and brothers of women with PCOS

**Fig. 9:**
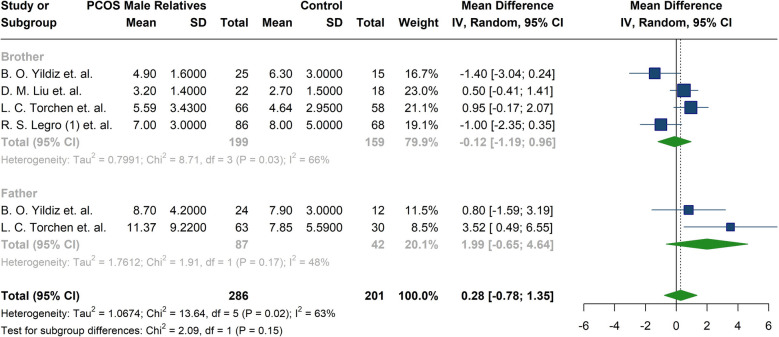
Forest plot of the subgroup analysis comparing Follicle-stimulating hormone (IU/L) between fathers and brothers of women with PCOS

Total testosterone, DHEAS, bioavailable testosterone, FSH, LH, and Androstenedione were reported in thirteen studies [19–21, 25–28, 32–34, 37, 38, 42]. The meta-analysis of hormonal profiles demonstrated 1.29 µmol/l higher levels of DHEAS in male relatives of patients diagnosed with PCOS compared to the controls, five studies reported significant differences between the two groups (MD: 1.29; 95%CI: [0.66; 1.92]; I^2^:42%) [20, 25, 26, 28, 33] (Fig. 7) (Table 4). In contrast, total testosterone levels in male relatives of women with PCOS, with only one study reporting a significant difference [26] (MD: −1.11; 95%CI: [−2.56; 0.34]; I^2^: 58%) (Fig. 7) (Table 4), This remained consistent after subgroup analysis for fathers and brothers (MD: −1.87; 95%CI: −5.48; 1.74; I^2^: 56%, for fathers and MD: −0.71; 95%CI: −2.16; 0.75; I^2^: 56%, for brothers) (Table 5) (Fig. 8). Furthermore, no notable differences were observed in the concentrations of bioavailable testosterone (MD: −0.28; 95%CI: [−1.77; 1.21]; I^2^:69%) or androstenedione (MD:0.05; 95%CI: [−0.67; 0.78]; I^2^:57%) between male relatives of individuals diagnosed with PCOS and male control subjects (Fig. 7) (Table 4). Only one study, R. Krysiak et al., reported significant differences in both androstenedione and bioavailable testosterone levels [34]. Similarly, no significant differences were observed in FSH levels (MD:0.47; 95%CI: −1.03; 1.96; I^2^:79%) (Fig. 7) (Table 4), even after subgroup analysis for fathers and brothers (MD:1.99; 95%CI: −0.65; 4.64; I^2^:48%, for fathers and MD: −0.12; 95%CI: −1.19; 0.96; I^2^:66%, for brothers) (Table 5) (Fig. 9). No significant differences were identified in the LH levels between the groups; except for one study reporting a significant difference [25] (MD: −0.40; 95%CI: [−1.33; 0.53]; I^2^:82%) (Fig. 7) (Table 4). Studies by Liu et al. [27] and Legro et al. [28] found no significant differences in LH, FSH, and total testosterone between brothers of women with PCOS and controls. Liu et al. [27] reported higher DHEAS in control males, while Legro et al. [28] reported significantly higher DHEAS levels in brothers of women with PCOS. Torchen et al. [21] reported significant differences in LH and FSH concentration between first-degree male relatives of women with PCOS and controls. However, Yildiz et al. [26], did not confirm differences in total testosterone. The investigation into total testosterone levels revealed no statistical differences across various studies [21, 25, 26, 28, 34]. In contrast, conflicting data were observed concerning DHEAS levels; Subramaniam et al. [33] and Lenarcik et al. [25] found significantly elevated DHEAS concentration in male siblings of women with PCOS, a previously noted association not corroborated by other studies [26, 27]. Regarding the androstenedione levels, Krysiak et al. [34] reported an important elevation in the experimental group of brothers; however, this finding was not validated by other research [25, 27]. Additionally, bioavailable testosterone levels were found to be elevated in brothers of women with PCOS compared to control men [34]; yet conflicting results were noted in the study by Legro et al. [28]. Torchen et al. [21] identified significant discrepancies in AMH levels in male relatives of women with PCOS in comparison to control males. In relation to neonatal androgen, Kollmann et al. [42] reported significantly higher androstenedione in boys born to women with PCOS compared to control boys. Nevertheless, no marked distinctions were observed in AMH and total testosterone between groups.

### CVD

**Fig. 10:**
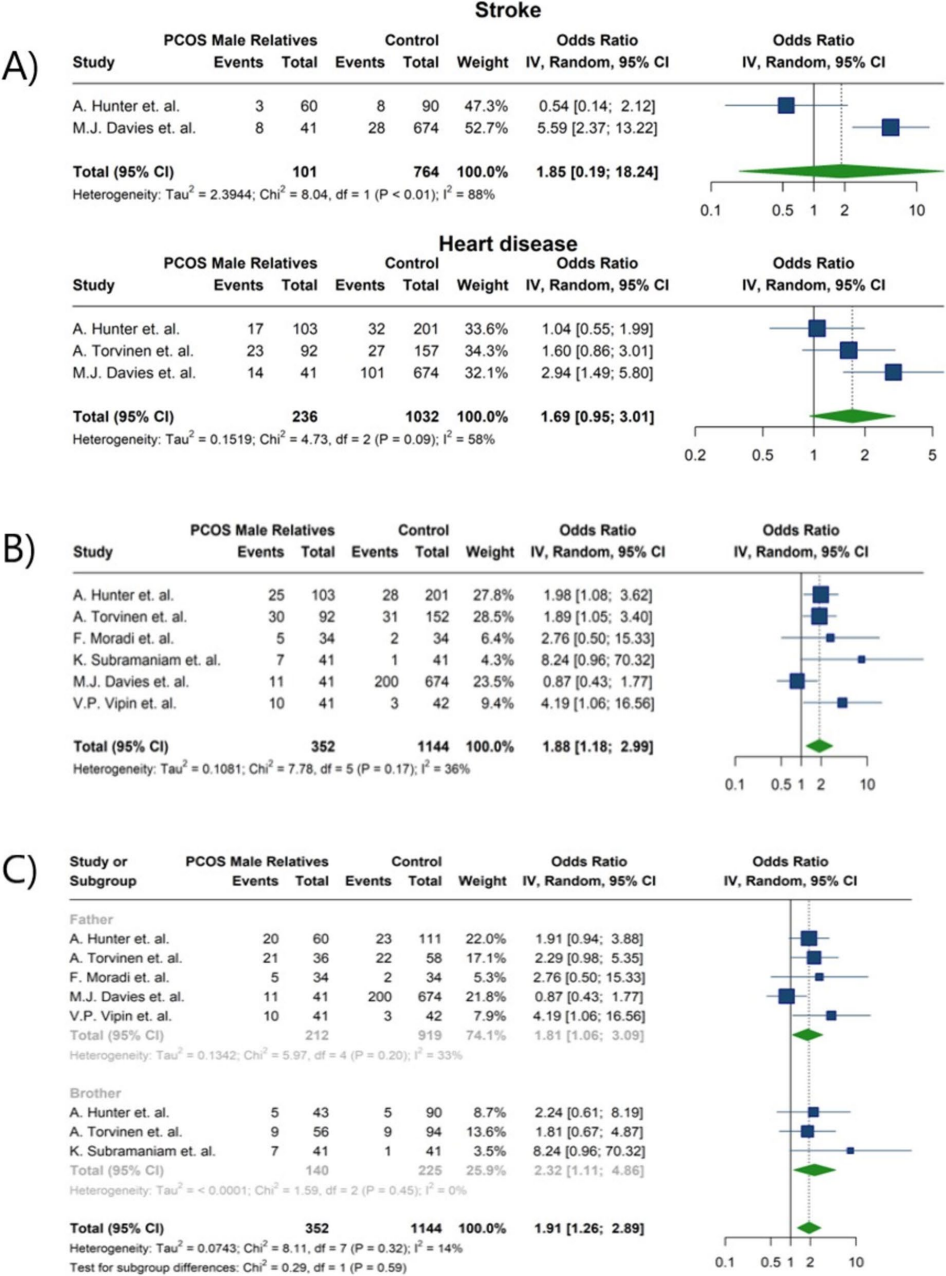
Forest plot illustrating the meta-analysis of the odds of cardiovascular diseases in male relatives of women with PCOS compared to control subjects. **A** Stroke or heart disease (as defined by figure title) **B**) Hypertension **C**) Forest plot of the subgroup analysis comparing prevalence of Hypertension between fathers and brothers of women with PCOS

Six studies compared CVD, stroke, and hypertension between two groups [22, 31–33, 36, 40]. A crucial association was disclosed between high blood pressure and male relatives of women with PCOS (OR:1.88; 95%CI: 1.18; 2.29; I^2^: 36%) (Fig. 4) (Table 4). The prevalence of hypertension is statistically enhanced in both brothers and fathers of patients with PCOS (OR:2.23; 95%CI: 1.11; 4.86; I^2^: 0%); OR: 1.81; 95%CI: 1.06; 3.09; I^2^: 33%, respectively) (Fig. 10) (Tables 4 and 5). Among six studies, half of them reported a significantly higher risk of hypertension in male relatives of women with PCOS [22, 36, 40]. In contrast, the prevalence of cardiovascular disease and stroke did not reach statistical significance between the two groups (OR:1.69; 95%CI: 0.95;3.01; I^2^: 58% and OR: 1.85; 95%CI: 0.19; 18.24; I^2^: 88%) (Fig. 10) (Table 4). In the investigation carried out by Hunter et al. [22] the prevalence of cardiovascular disease, stroke, chest pain, and various other medical conditions exhibited no statistically significant difference in brothers of PCOS probands, contrary to control males. However, the findings presented by Davis et al. [31] demonstrated that fathers of women afflicted with PCOS were more than twice as likely to have a diagnosis of cardiovascular disease and over four times more likely to have experienced a stroke. Although in the research undertaken by Taylor et.al. [35] an elevated prevalence of heart attack and stroke was documented. Regardless of various studies that have indicated a higher prevalence of hypertension in male relatives of women with PCOS [33, 41, 46]Other investigations have presented findings that are inconsistent with these results [22, 31].

### Baldness

**Fig. 11:**
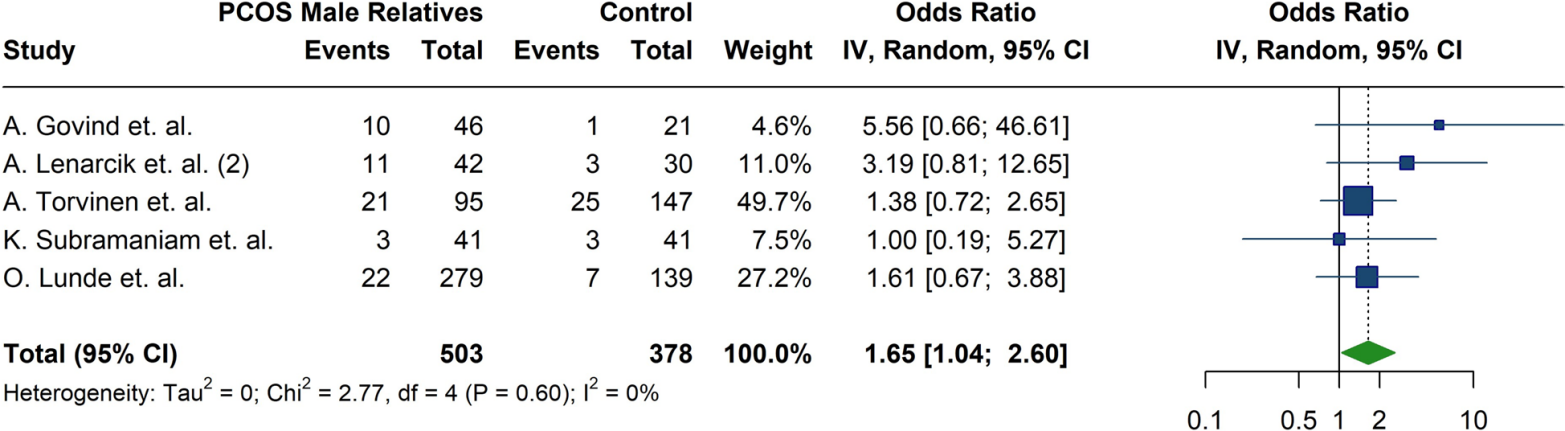
Forest plot illustrating the meta-analysis of the odds of baldness in male relatives of women with PCOS compared to control subjects

The results of our meta-analysis showed a significantly higher likelihood of baldness in male relatives of women with PCOS compared to controls (OR:1.65; 95%CI: [1.04; 2.60]; I^2^: 0%) (Fig. 11) (Table 4). Five studies were included in this meta-analysis [18, 25, 30, 33, 40]. Regarding baldness, Lunde et. al [30] observed a higher prevalence of baldness among first-degree relatives of women with PCOS, whereas other studies did not confirm this association finding [25, 33].

## Discussion

In this systematic review and meta-analysis, we synthesized findings from studies examining various aspects of the male equivalent of PCOS in first-degree male relatives of women with PCOS. Our analysis revealed that these male relatives were more likely to have obesity and higher BMI values. Although they did not show increased rates of diabetes, they had significantly higher FBS and HOMA-IR levels. Lipid profiles showed higher levels of LDL-C, TG, and total cholesterol, while HDL-C levels were similar to controls. Additionally, the prevalence of hypertension was higher among male relatives, although no significant differences were observed in stroke or heart disease. Hyperandrogenic features were also present, shown by a higher prevalence of androgenetic alopecia and elevated DHEAS levels. No significant differences were detected in LH, FSH, or other testosterone measures.

Although the underlying causes of PCOS and, by extension, its male equivalent, are complex and not yet fully understood, genetic factors appear to play a central role, with heritability estimated at approximately 70% [47]; male relatives of women with PCOS may inherit the same genes that contribute to PCOS risk in females [13]. The broad biochemical, endocrine, metabolic, and clinical manifestations of PCOS suggest that multiple genetic factors contribute to the pathogenesis of its various phenotypes. Candidate genes include those involved in steroid production, androgen receptor function, and insulin signaling. Genome-wide association studies (GWAS) have identified HMGA2, THADA, and INSR as significant contributors to PCOS [48]. Notably, these genes are also linked to T2DM [45]. The FBP1 gene, part of the insulin signaling pathway, has also been implicated in the pathogenesis of PCOS [48]. Our findings suggest that this genetic involvement extends to male relatives of individuals with PCOS, shown by their higher FBS levels and insulin resistance compared to controls. FBP1 plays a critical role in gluconeogenesis by catalyzing the conversion of fructose-1,6-bisphosphate to fructose-6-phosphate, a key step in glucose metabolism. Dysregulation of this pathway may result in impaired glucose homeostasis, a feature commonly observed in both women with PCOS and their male relatives. Furthermore, insulin has been shown to play an important role in regulating hair follicle growth (anagen) and resting (telogen) cycles [49]. Consequently, PCOS-associated insulin resistance may contribute to hair loss, similar to that observed in diabetes [50]. A meta-analysis has linked the cytochrome P450 family 17 (CYP17) gene to PCOS susceptibility, with one allele of the gene also associated with male pattern baldness [44]. The CYP17, encoding the 17-α-hydroxylase and 17,20 lyase enzymes, plays a crucial role in the androgen biosynthesis pathway in both sexes [51]. In men, various polymorphisms of CYP17 have been associated with bioavailable testosterone levels, bone size [43], risk of prostate cancer [52]. It is thus possible that CYP17 also contributes to the increased level of DHEAS observed in the male relatives of PCOS patients [53] Lastly, DENND1A [54], YAP1 [55], ERBB3, and RAB5B [48, 56] are also implicated in PCOS, and have been shown to be involved in endocytosis, cellular trafficking calcium signaling, and the ERBB3 signaling pathway [57–59] As these genes regulate insulin metabolism and hormone production, their involvement may contribute to the higher rate of biochemical and metabolic abnormalities in both PCOS women and their male relatives.

PCOS is widely recognized as a condition with a strong heritable component, which has led to extensive investigation into the phenotypic expression of hyperandrogenism and metabolic abnormalities among first-degree relatives of affected women. Research in this area aims to explain the genetic and biological mechanisms of PCOS by studying how common and how severe related hormone and metabolic problems are in families. Growing evidence suggests that PCOS is not solely a female-specific condition but also has family and possible transgenerational effects. Male first-degree relatives, as well as female relatives, have been found to exhibit hormonal and metabolic abnormalities that are not gender specific. A meta-analysis reported that relatives of women with PCOS had a significantly higher prevalence of metabolic syndrome, hypertension, and dyslipidemia. Specifically, mothers, fathers, and sisters exhibited an increased risk of metabolic syndrome; fathers and sisters had a higher risk of hypertension; and brothers and fathers showed a higher prevalence of dyslipidemia. Systolic blood pressure, total cholesterol, low-density lipoprotein cholesterol, and triglyceride levels were all significantly higher in first-degree relatives compared to controls [41]. Furthermore, a 2018 meta-analysis examining the risk of diabetes and insulin resistance reported that the prevalence of type 2 diabetes was significantly higher in the mothers and fathers of women with PCOS. Fasting insulin levels and HOMA-IR were also significantly elevated in both the parents and siblings of affected women. Although sisters and brothers of women with PCOS exhibited a higher prevalence of type 2 diabetes, these differences did not reach statistical significance [60]. Additionally, a 2022 systematic review examining the hormonal profiles and reproductive health of relatives of women with PCOS reported a higher prevalence of PCOS, menstrual irregularities, and ovarian morphological changes among female first-degree relatives. These relatives also exhibited elevated LH, total testosterone, unconjugated testosterone, free androgen index, DHEAS, and AMH. Subgroup analyses indicated that some of these hormonal alterations emerged during puberty. Among male first-degree relatives, fathers of women with PCOS were found to have an increased risk of premature androgenetic alopecia, and elevated DHEAS levels were observed [61].

It is important to note that the adverse effects of PCOS are not confined to parents or siblings. A 2020 systematic review examining the cardiometabolic health of offspring of women with PCOS reported that these offspring exhibited higher insulin resistance and HDL levels, but lower birth weight compared to controls. Sex-specific differences were also observed: female offspring demonstrated higher 2-h fasting insulin levels and greater HDL differences than males, whereas differences in LDL-C and total cholesterol were smaller in females [62]. Additionally, more recent studies have indicated that male offspring of mothers with maternal hyperandrogenism are at an increased risk of prediabetes. However, no significant differences were observed in the risk of type 2 diabetes, prehypertension, hypertension, dyslipidemia, overweight, or obesity [63, 64]. In contrast, female offspring of mothers with PCOS have been shown to be at an increased risk of type 2 diabetes mellitus, overweight, and metabolic syndrome [65, 66]

Male relatives of women with PCOS showed elevated DHEAS levels, potentially indicating a shared underlying abnormality in steroid hormone synthesis across sexes [28]. As discussed above, polymorphisms of the CYP17 gene may play a role in this increased serum level. One implication of elevated DHEAS levels in men is the increased chance of hepatic steatosis, a hepatic manifestation of the metabolic syndrome [67]. Additionally, through peripheral conversion by steroid sulfatase, DHEAS is ultimately metabolized to dihydrotestosterone in target tissues, including hair follicles. This pathway may underlie traits such as androgenic alopecia, as plasma DHEAS levels correlate with balding in young men [68]. Furthermore, while elevated DHEAS has also been associated with reduced obesity and smaller abdominal fat accumulation, suggesting a potentially favorable effect on some metabolic parameters, its cardiovascular effects are inconsistent. Large population studies and intervention trials have generally failed to demonstrate a protective effect of DHEAS on cardiovascular endpoints, highlighting that any favorable impact on obesity or fat distribution does not necessarily translate into meaningful reductions in cardiovascular risk [69]. Collectively, these findings suggest that higher DHEAS in male relatives of women with PCOS may confer a mixed endocrine and metabolic profile.

This study has both limitations and strengths. To our knowledge, it is the first systematic review to provide a detailed evaluation of the male equivalent of PCOS by systematically examining its diverse metabolic, biochemical, and phenotypic manifestations. Furthermore, in cases of substantial heterogeneity among studies, we conducted subgroup analyses stratified by family relationship, separating brothers and fathers of affected women. This approach allowed us to explore potential differences in the expression of PCOS-related traits between these groups, providing more detailed insights into the variability, inheritance patterns, and mechanisms underlying the male phenotype associated with PCOS.

### Limitations of the study

Our study has several limitations. First, a high degree of heterogeneity was observed in several analyses, which could not be fully explained by subgroup analysis based on the type of male relative. This heterogeneity may reflect differences in baseline characteristics, including age, ethnicity, or BMI, as well as methodological differences such as sampling, study design, and the diagnostic criteria (such as NIH and Rotterdam criteria) for female PCOS probands in the original studies. These factors may affect study outcomes; further rigorous investigations are warranted to explain the sources of heterogeneity.

Additionally, some outcomes assessed in this review were derived from a limited number of studies or small sample sizes, restricting the statistical power and robustness of the conclusions. Larger, well-designed studies are needed to provide more solid and generalizable evidence.

Finally, most included studies did not consider lifestyle factors, such as diet, physical activity, or other behaviors, particularly for outcomes like obesity and metabolic disturbances. Future research should include adjustments for these lifestyle factors to better isolate the genetic and physiological contributions to the male equivalent of PCOS and to enhance the accuracy and validity of findings.

Screening male relatives for metabolic and hormonal risk factors may help identify at-risk individuals and inform preventive interventions. These assessments could include evaluation of blood lipids, blood glucose, blood pressure, hormonal profile and early signs of hair loss. If abnormalities are detected, tailored management strategies could be considered, potentially differing from those for individuals without a family history of PCOS. Implementing such measures could help translate our research findings into clinical practice, enabling earlier detection and intervention, and guiding future studies on preventive strategies in high-risk populations.

## Conclusion

In conclusion, this systematic review and meta-analysis provide strong evidence for a male equivalent of PCOS, shown by consistent changes in biochemical profiles, hormonal levels, and the increased prevalence of metabolic and cardiovascular comorbidities among fathers and brothers of women with PCOS. Male relatives were more likely to have obesity, hypertension, and androgenetic alopecia, as well as a higher risk of dyslipidemia and insulin resistance. Since these features mirror those seen in women with PCOS, they are likely due to shared genetic causes. To confirm these findings and better define other clinical signs in male relatives, there is a need for large, well-designed studies with strong methods.

## Data Availability

All data has been presented in the manuscript.

## Abbreviations

PCOS: Polycystic ovary syndrome
MD: Mean difference
CI: Confidence interval
BMI: Body mass index
DHEAS: Dehydroepiandrosterone sulfate
OR: Odds ratio
NIH: National Institutes of Health
AGA: Androgenetic alopecia
LH: Luteinizing hormone
FSH: Follicle-stimulating hormone
SHBG: Sex hormone-binding globulin
PRISMA: Preferred Reporting Items for Systematic Reviews and Meta-Analyses
IRHRC: Infertility and Reproductive Health Research Center
AMH: Anti-Müllerian hormone
HDL-C: High-density lipoprotein cholesterol
LDL-C: Low-density lipoprotein cholesterol
HOMA-IR: Homeostatic Model Assessment of Insulin Resistance
uT: Urinary testosterone
NOS: Newcastle–Ottawa Scale
R: R statistical software
FBS: Fasting blood sugar
QUICKI: Quantitative Insulin Sensitivity Check Index
TG: Triglycerides
CVD: Cardiovascular disease
GWAS: Genome-wide association studies
T2DM: Type 2 diabetes mellitus
FBP1: Fructose-1,6-bisphosphatase 1
CYP17: Cytochrome P450 family 17
